# Fever and Pain Management in Childhood: Healthcare Providers’ and Parents’ Adherence to Current Recommendations

**DOI:** 10.3390/ijerph13050499

**Published:** 2016-05-13

**Authors:** Genny Raffaeli, Annalisa Orenti, Monia Gambino, Walter Peves Rios, Samantha Bosis, Sonia Bianchini, Claudia Tagliabue, Susanna Esposito

**Affiliations:** Pediatric Highly Intensive Care Unit, Department of Pathophysiology and Transplantation, Università degli Studi di Milano, Fondazione IRCCS Ca’ Granda Ospedale Maggiore Policlinico, Via Commenda 9, Milano 20122, Italy; genny.raffaeli@gmail.com (G.R.); annalisa.orenti@guest.unimi.it (A.O.); monia.85@hotmail.it (M.G.); walterpeves@gmail.com (W.P.R.); samantha.bosis@gmail.com (S.B.); bianchini.sonia@fastwebnet.it (S.B.); hollie1979@yahoo.it (C.T.)

**Keywords:** analgesics, antipyretics, fever, ibuprofen, pain, paracetamol

## Abstract

In order to evaluate the adherence of healthcare providers and parents to the current recommendations concerning fever and pain management, randomized samples of 500 healthcare providers caring for children and 500 families were asked to complete an anonymous questionnaire. The 378 health care providers (HCPs) responding to the survey (75.6%) included 144 primary care pediatricians (38.1%), 98 hospital pediatricians (25.9%), 62 pediatric residents (16.4%), and 71 pediatric nurses (19.6%); the 464 responding parents (92.8%) included 175 whose youngest (or only) child was ≤5 years old (37.7%), 175 whose youngest (or only) child was aged 6–10 years (37.7%), and 114 whose youngest (or only) child was aged 11–14 years (24.6%). There were gaps in the knowledge of both healthcare providers and parents. Global adherence to the guidelines was lower among the pediatric nurses than the other healthcare providers (odds ratio 0.875; 95% confidence interval 0.795–0.964). Among the parents, those of children aged 6–10 and 11–14 years old, those who were older, and those without a degree answered the questions correctly significantly less frequently than the others. These findings suggest that there is an urgent need to improve the dissemination of the current recommendations concerning fever and pain management among healthcare providers and parents in order to avoid mistaken and sometimes risky attitudes, common therapeutic errors, and the unnecessary overloading of emergency department resources. Pediatric nurses and parents with older children, those who are older, and those with a lower educational level should be the priority targets of educational programmes.

## 1. Introduction

Fever and pain are frequent in infants and children of all ages, and represent more than 30% of all of the complaints referred to pediatricians [1,2,3]. It is well known that fever is part of the body’s natural response to infections and inflammatory or immunological disorders [4]. As it is still a cause of considerable concern for health care providers (HCPs) and parents [1,5,6], efforts have been made to provide guidelines for its management throughout the world [2,7]. For example, the Italian Society of Pediatrics has recently updated its 2009 Italian Fever Guideline (an evidence-based document on fever management in childhood) [8,9], but, despite a national dissemination campaign of conferences, courses, and web-based strategies, surveys of small convenience samples of Italian pediatricians have shown that there is still considerable variability in approaches that lead to erroneous medical and parental behaviours [10,11,12].

Pediatric pain is mainly experienced in association with an illness or injury, and there is growing concern about its potentially adverse long-term effects on the developing brain [13,14,15,16]. Although various standardised means of pain assessment and treatment have been proposed [15,17], it has been shown that too many children still experience under-recognised and under-treated pain [17]. The aim of this survey was to evaluate views and practices of HCPs and children’s parents, and their adherence to the current recommendations.

## 2. Methods

### 2.1. Study Design and Study Population

This cross-sectional study was carried out between 15 November 2014 and 15 February 2015, after the protocol had been approved by the Ethics Committee of the Fondazione IRCCS Ca’ Granda Ospedale Maggiore Policlinico, Milan, Italy (Project identification code: 850-2014). The written informed consent of the HCPs or parents who completed the questionnaire was obtained.

A random sample of 500 HCPs involved in pediatric care (primary care pediatricians, hospital pediatricians, residents in pediatrics, and pediatric nurses) in Lombardy, Italy, and 500 families with children aged <15 years living in the same region but not necessarily patients of the random sample of HCPs were invited to participate in the survey by means of an e-mail sent approximately 30 days before the beginning of the study. HCPs were selected using a computer-based list among those living and working in Lombardy Region who attended the 33rd National Congress of Antibiotic Therapy in Pediatric Age who was held in Milan, Italy, from 29 October 2014 to 31 October 2014. Families were selected using a computer-based list among those who attended the Children’s Outpatient Clinic of the Fondazione IRCCS Ca’ Granda Ospedale Maggiore Policlinico, Milan, Italy, from 1 September 2014 to 31 October 2014; the inclusion criteria included an adequate knowledge of Italian and exclusion criteria the presence of siblings with chronic diseases. A monthly reminder was sent in order to encourage the HCPs and parents to answer the questionnaire, which was eventually completed by 378 HCPs (75.6%) and 464 parents (92.8%). The monthly reminder by e-mail was the only used strategy for encouraging participation; no compensation was provided to HCPs and families who filled in the questionnaire. The high response rate to the survey among families could be explained by the fact that the study was proposed by the Outpatient Clinic that regularly followed their children and that is the main Pediatric Clinic in Lombardy Region.

### 2.2. Questionnaires

Each group was administered a different anonymous questionnaire consisting of three main sections (one for personal and demographic data, the second dealing with the management of fever, and the third with the management of pain), and adapted to the respondents in terms of content and terminology. Questionnaires were developed according to criteria described by Boynton *et al.* [18]. The multiple-choice questions, which were prepared by a pediatrician expert in pediatric infectious diseases (Susanna Esposito) and tested on a pilot sample of 20 HCPs and 20 parents) reflected the revised Italian guideline for fever management [9] and guidance on pain control [17]. All the questions and possible answers are included in Table 1, Table 2, Table 3, Table 4, Table 5 and Table 6. The pilot sample enrolled for the validation procedure was not included in the random sample considered in the final statistical analysis. The questionnaire was written in Italian and it was translated in English for this publication by an English native speaker.

### 2.3. Statistical Analysis

The number and corresponding percentage of the occurrence of responses to each question were recorded and compared among groups of HCPs and among groups of parents using chi squared test corrected for the presence of multiple responses for subject [19]. The median value and range of age were recorded and their distribution was compared among groups of HCPs and among groups of parents using a two-sided Kruskal Wallis test after checking that they were normally distributed by means of the Shapiro-Wilk statistic. A *p*-value < 0.05 was considered statistically significant.

With regards to HCPs, comparisons were performed among primary care paediatricians, hospital paediatricians, residents in paediatrics, and pediatric nurses. With regards to parents, comparisons were performed among those with at least one sibling ≤5 years old, 6–10 years old and 11–14 years old. In case of more than one child, the age of the youngest child was considered.

Poisson’s regression analysis was used to evaluate the associations between the number of correct answers according to the Italian guidelines and background data (*i.e.*, gender, age, and role in the HCPs’ group and gender, parents’ age, child’s age, and education in parents’ group). The associations between correct answers to the individual questions and the participants’ background data (all used for correction) were assessed by means of multiple logistic regression analysis with covariates additive effects. All of the statistical analyses were made using R software version 3.1.1 (R Development Core Team, Vienna, Austria), with the package multiple response categorical variable (MRCV) added.

## 3. Results

### 3.1. Questionnaire Administered to Healthcare Providers (HCPs)

Table 1 shows the demographic characteristics of the 378 responding HCPs: 144 primary care pediatricians (38.1%); 98 pediatricians who worked in hospitals (25.9%); 62 pediatric residents (16.4%); and 71 pediatric nurses who worked in hospitals (19.6%). The majority (297/378, 78.57%) were women, and worked in a principal city (241/378, 63.76%). Median age was significantly different among groups of HCPs (*p* < 0.001), whereas gender distribution was similar in all of the groups.

A higher percentage of residents and nurses worked in a principal city hospital in comparison with primary care paediatricians and hospital paediatricians (90.32% and 78.38% *vs.* 47.22% and 60.2% respectively). Overall, 33.7% of hospital paediatricians and 29.9% of primary care paediatrician had an additional medical specialty other than paediatrics, whereas the corresponding percentage among residents and nurses were very low (4.8% and 2.7%). Table 2 shows the HCPs’ attitudes towards and knowledge of fever management. A higher percentage of residents and nurses recommended the use of an electronic thermometer and evaluation of axillary temperature, and a higher percentage of hospital pediatricians defined fever as an axillary temperature of ≥37.9 °C (although many of the participants in all of the groups wrongly answered this question). Most of the respondents (311/378, 82.28%) prescribed oral paracetamol as the first-line antipyretic but, in comparison with the other groups, the pediatric nurses recommended oral paracetamol less frequently, and many of them recommended rectal paracetamol. The majority of respondents who prescribed paracetamol as the first-choice drug did so because it is better tolerated than ibuprofen, whereas the main reason for prescribing ibuprofen was because it is considered more efficacious than paracetamol and substantially similarly tolerated. Many of the respondents did not know the recommended childhood doses of paracetamol and ibuprofen, with more pediatric nurses giving wrong answers to both questions. A minority of respondents recommended alternating paracetamol and ibuprofen (58/378, 15.34%), with no difference between the groups. In terms of drug safety, pediatric nurses were less frequently aware that the main paracetamol-related adverse event is hepatotoxicity and that the main ibuprofen-related adverse event is gastric involvement.

Table 3 summarises how the HCPs managed pediatric pain. In comparison with all of the other groups, fewer pediatric residents and primary care paediatricians tried to quantify pain in comparison with nurses and hospital pediatricians. Most of the respondents (347/378, 91.8%) knew how to quantify pain in an infant, but fewer (260/378, 68.78%) knew how to do so in a child aged 3–4 years. However, this was known by more residents than the members of the other groups, and more residents also knew that pain management is related to pain intensity (*p* = 0.036), and prescribed paracetamol for mild-to-moderate pain. Nevertheless, paracetamol was considered the first-line drug for managing this type of pain by most of the respondents as a whole (332/378, 87.83%).

The Poisson regression analysis showed that global adherence to the guidelines was lower among the pediatric nurses than among the primary care paediatricians (odds ratio (OR) 0.875; 95% confidence interval (CI) 0.795–0.964), whereas there was no significant difference between the other two groups. Furthermore, there was no relationship between adherence and age, gender, the number of years of practice, or working location.

The associations between the correct answers to individual questions and the participants’ baseline data, which were assessed by means of multiple logistic regression analysis with covariates additive effects, showed that, in comparison with primary care pediatricians, pediatric nurses correctly answered the questions related to the recommended drug for fever management (OR 0.272; 95% CI 0.137–0.542), paracetamol dosage (OR 0.283; 95% CI 0.144–0.555), and the main adverse event associated with ibuprofen (OR 0.477; 95% CI 0.248–0.918) significantly less frequently, and that hospital pediatricians quantified pain significantly more frequently (OR 5.0003; 95% CI 1.043–24.004). Interestingly, increasing age significantly decreased the probability of knowing that hepatotoxicity was the main paracetamol–related adverse event (OR 0.947; 95% CI 0.916–0.979) and how to quantify pain in children aged 3–4 years (OR 0.928; 95% CI 0.928–0.977).

### 3.2. Questionnaire Administered to Parents

Table 4 shows the demographic characteristics of the 464 responding parents: 175 whose youngest or only child was ≤5 years old (37.7%), 175 whose youngest or only child was aged 6–10 years (37.7%), and 114 whose youngest or only child was aged 11–14 years (24.6%). The majority were Caucasian (433/464, 93.32%), mothers (373/464, 80.39%), had one child (251/464, 66.58%), and lived in a provincial capital (309/464, 66.59%). Parents age was significantly different among groups (*p* < 0.001), with increasing parents age at increasing children age. Parents of children ≤5 years old had more often only one sibling. The remaining demographic characteristics were no statistically different among different groups.

Table 5 shows parental fever management. In comparison with the other groups, the parents whose youngest or only child was ≤5 years old were more aware that an electronic thermometer is the recommended means of measuring childhood temperature, more frequently consulted a pediatrician in the case of a febrile child, less frequently used physical means to reduce fever, more frequently knew that the main ibuprofen-related adverse event is gastric involvement, less frequently knew that fever should be evaluated by means of axillary temperature, more frequently preferred the rectal administration of paracetamol, and more frequently alternated the administration of paracetamol and ibuprofen. Paracetamol was the most frequently prescribed antipyretic in all of the groups (409/464, 88.15%), but the minority of the parents who used paracetamol (112/409, 27.38%) knew that it is potentially toxic, and only 60/409 (14.67%) knew that the main paracetamol-related adverse event is hepatotoxicity. The families had the possibility to request by e-mail a clarification for medical terms if unclear, but nobody asked a clarification.

Table 6 shows how the parents considered and managed their children’s pain. Most of them (235/464, 50.65%) considered pain educational, knew how to recognise it (324/464, 69.83%), and used paracetamol as the first-line drug to manage mild-to-moderate pain (363/464, 78.23%). Also for this section, the families had the possibility to request by e-mail a clarification for medical terms if unclear, but nobody asked a clarification.

The Poisson regression analysis showed that the parents’ knowledge of the guidelines concerning the management of fever and pain was not significantly influenced by their age, gender or education, or the age of their children. The associations between correct answers to the individual questions and the parents’ baseline data, which were assessed by means of multiple logistic regression analysis with covariates additive effects, showed that the question concerning which thermometer should be used to measure fever was correctly answered significantly less frequently by the parents whose youngest or only child was 6–10 (OR 0.438 and 95% CI 0.264–0.727) or 11–14 years old (OR 0.435 and 95% CI 0.226–0.835) compared to parents of children ≤5 years, as was the question concerning the alternation of paracetamol and ibuprofen (OR respectively 0.31 (95% CI 0.143–0.673) and 0.278 (95% CI 0.103–0.748)); however, the same groups significantly more frequently correctly answered the question concerning the site of measuring fever (OR respectively 3.91 (95% CI 2.35–6.505) and 10.188 (95% CI 4.578–22.674)). The parents whose youngest or only child was 11–14 years old also significantly less frequently correctly answered the question concerning the need to consult a pediatrician before administering an antipyretic (OR 0.381; 95% CI 0.196–0.74) and the use of physical means in addition to antipyretics to reduce fever (OR 0.434 and 95% CI 0.231–0.815). Older age was associated with fewer correct answers to the question concerning the thermometer that should be used to measure fever (OR 0.947; 95% CI 0.907–0.989). The parents who had a degree correctly answered the question concerning the need to consult a pediatrician when a child has fever significantly less frequently (OR 0.318; 95% CI 0.213–0.475), but correctly answered the questions concerning the first approach to fever (OR 1.471; 95% CI 1.006–2.151) and the preferred route of administering of antipyretics (OR 1.772; 95% CI 1.148–2.735) significantly more frequently.

## 4. Discussion

The findings of this study show that the attitudes of HCPs and parents in management of childhood fever and pain are not in line with current recommendations. Interestingly, they also show that the main educational interventions should concentrate on pediatric nurses and older parents, and those with older children or a lower educational level.

Like those of previous studies [3,5,10,20], our data indicate that neither the HCPs nor the parents had a clear idea as to how to define fever and, although the majority of parents considered it a defence mechanism, a substantial number considered it a sign of disease that should only be eliminated if high. In order to improve their approach to this clinical sign, parents need to be clearly informed that it has been extensively proved that the main goal when treating a feverish child is to reduce illness-related pain or discomfort (*i.e.*, sore throat associated with pharyngotonsillitis) [21].

In line with the pediatric guidelines [8,9,17,22], most of the HCPs and parents recommended the use of paracetamol as the first-line drug for treating fever and mild-to-moderate pain. However, the route of administration and dose were not appropriate in a number of cases: as found in other studies [10], many nurses and parents preferred rectal administration over the recommended oral administration, and there was considerable variability in the dose recommended by HCPs, whereas De Martino *et al.* have recently shown that optimal efficacy and safety can be achieved with an oral dose of 15 mg/kg every 4–6 h [23]. This finding is quite interesting both for designing further studies assessing compliance to different formulations as well as for manufacturers in order to focus their attention on the preferred formulations. Furthermore, the HCPs often prescribe a higher than recommended dose of ibuprofen, the alternative to paracetamol [24], thus underlining the risk of drug misuse [25]. A Scottish study has recently found similar misdosing and suggested that many prescriptions by general practitioners contain errors [26]. Furthermore, only a minority of our parents were adequately informed about paracetamol-associated adverse events. Furthermore, it has been shown that paracetamol is one of the four medications most frequently involved in pediatric overdosing [27]. Regarding ibuprofen, it is important to highlight that although gastritis and gastrointestinal bleeding are the most frequent side effects, renal insufficiency may be the most severe side effect [28].

Some HCPs and parents recommended the alternate administration of paracetamol and ibuprofen as a means of reducing fever despite the fact that it has been shown to be less safe and not more efficacious [29,30]: furthermore, the majority of parents still use physical means as a first step in reducing fever despite the lack of evidence that it leads to any benefit [9,21]. The proportion of parents that stated to use physical means is extremely high and deserves attention for educational programmes.

Our findings suggest that pediatric pain might be under-recognised and consequently undertreated but, in comparison with a similar Canadian survey [31], revealed an improvement in overall pain management. Although some questions do not have really mutually exclusive answers and the choice of the analgesis drug should be driven by different aspects, parental understanding of the long-term consequences and neurodevelopmental outcomes of pediatric pain seems still inadequate.

Interestingly, the results of the Poisson regression and multiple regression analyses of the HCPs showed that pediatric nurses knew least about fever and pain management. Given their continuous contact with children during hospitalisation and the fact that they manage the administration of antipyretics and analgesics, this indicates that they should be the primary target of educational programmes.

The multiple regression analyses of the parents showed that those with older children tend to answer less correctly than those with younger children. Parental educational level seems to play a positive role in improving concordance with the guidelines. In the case of fever, the parents with a higher educational level seemed to be more prepared and more ready to administer the right drug using the correct route of administration. However, as they tend to act independently without consulting their pediatrician, they run a greater risk of drug misdosing.

Overall, our findings indicate that there is still a need to improve the knowledge of HCPs and parents concerning the management of fever and pain management not only to guarantee children’s health, but also to reduce the unnecessary overloading of pediatric emergency departments. There is therefore a need for educational interventions and adequate counseling, which should mainly be aimed at pediatric nurses and older and less educated parents, and those with older children, and be followed by further studies to evaluate their effectiveness.

The main limitations of this study are that it is based on self-reported evaluations rather than observed real life practice [20], we did not use questions derived from previous studies, survey validation was performed in a limited sample of HCPs and families, the questionnaire was performed in HCPs and families living in only one Italian Region, and no answer was provided to a part of questions. However, the results obtained were quite similar in the two groups of respondents and seem to be useful for preparing and implementing educational programmes. In addition, replication of this study appears possible in other settings where guidelines for the care of febrile children and children with pain are available in order to optimize educational programmes on these topics.

## 5. Conclusions

The findings of our survey suggest that there is an urgent need to improve the dissemination of the current recommendations concerning the management of fever and pain among HCPs and parents in order to avoid mistaken and sometimes risky attitudes, common therapeutic errors, and the unnecessary overloading of emergency department resources. Pediatric nurses and parents with older children, those who are older, and those with a lower educational level should be the priority targets of educational programmes.

## Figures and Tables

**Table 1:** Demographic data of responding healthcare providers.

Data	Finding	Total *n* = 378	Primary Care Paediatricians *n* = 144	Hospital Paediatricians *n* = 98	Pediatric Residents *n* = 62	Pediatric Nurses *n* = 74	*p*-Value
		*n* (%)	*n* (%)	*n* (%)	*n* (%)	*n* (%)	
Median age (min–max)		47 (24–80)	55 (30–80)	49 (31–76)	29 (25–37)	43 (24–64)	<0.001
Gender	Male	77 (20.37)	34 (23.61)	22 (22.45)	12 (19.35)	9 (12.16)	0.219
	Female	297 (78.57)	110 (76.39)	75 (76.53)	47 (75.81)	65 (87.84)	
	NA	4 (1.06)	0 (0)	1 (1.02)	3 (4.84)	0 (0)	
Where do you work?	Principal city	241 (63.76)	68 (47.22)	59 (60.2)	56 (90.32)	58 (78.38)	<0.001
	Industrial municipality	94 (24.87)	44 (30.56)	33 (33.67)	6 (9.68)	11 (14.86)	
	Rural municipality	39 (10.32)	32 (22.22)	5 (5.1)	0 (0)	2 (2.7)	
	NA	4 (1.06)	0 (0)	1 (1.02)	0 (0)	3 (4.05)	
Are you specialised in pediatrics?	No	136 (35.98)	0 (0)	0 (0)	62 (100.0)	74 (100.0)	Not applicable
	Yes	242 (64.02)	144 (100.0)	98 (100.0)	0 (0)	0 (0)	
Medical specialty other than paediatrics	None	255 (67.46)	101 (70.14)	61 (62.24)	53 (85.48)	40 (54.05)	0.046
	Allergology	8 (2.12)	2 (1.39)	4 (4.08)	0 (0)	2 (2.7)	
	Infectious diseases	9 (2.38)	1 (0.69)	7 (7.14)	1 (1.61)	0 (0)	
	Neonatology	22 (5.82)	13 (9.03)	9 (9.18)	0 (0)	0 (0)	
	Infant care	7 (1.85)	7 (4.86)	0 (0)	0 (0)	0 (0)	
	Nutrition	4 (1.06)	4 (2.78)	0 (0)	0 (0)	0 (0)	
	Hygiene	4 (1.06)	1 (0.69)	3 (3.06)	0 (0)	0 (0)	
	Others	27 (7.14)	15 (10.42)	10 (10.2)	2 (3.23)	0 (0)	
	NA	51 (13.49)	8 (5.56)	5 (5.1)	6 (9.68)	32 (43.24)	

In the case of the categorical variables, the table shows absolute frequencies with percentage frequencies in brackets. NA: not available.

**Table 2:** Fever management by healthcare providers.

Question	Multiple Choice Answers	Total *n* = 378	Primary care Pediatricians *n* = 144	Hospital Pediatricians *n* = 98	Pediatric Residents *n* = 62	Pediatric Nurses *n* = 74	*p*-Value
		*n* (%)	*n* (%)	*n* (%)	*n* (%)	*n* (%)	
What type of thermometer do you recommend to your patients?	Mercury	70 (18.52)	29 (20.14)	26 (26.53)	4 (6.45)	11 (14.86)	**0.004**
	Infra-red	24 (6.35)	11 (7.64)	5 (5.1)	2 (3.23)	6 (8.11)	
	Liquid crystals	33 (8.73)	19 (13.19)	8 (8.16)	3 (4.84)	3 (4.05)	
	**Electronic**	**267 (70.63)**	**99 (68.75)**	**61 (62.24)**	**53 (85.48)**	**54 (72.97)**	
	Gallium rectal thermometer	4 (1.06)	3 (2.08)	1 (1.02)	0 (0)	0 (0)	
	NA	18 (4.76)	8 (5.56)	9 (9.18)	1 (1.61)	0 (0)	
What site do you suggest for measuring a child’s temperature?	Rectum	99 (26.19)	61 (42.36)	24 (24.49)	1 (1.61)	13 (17.57)	**<0.001**
	**Armpit**	**266 (70.37)**	**94 (65.28)**	**65 (66.33)**	**50 (80.65)**	**57 (77.03)**	
	Ear	34 (8.99)	9 (6.25)	13 (13.27)	10 (16.13)	2 (2.7)	
	Forehead	10 (2.65)	3 (2.08)	1 (1.02)	2 (3.23)	4 (5.41)	
	Groin	2 (0.53)	2 (1.39)	0 (0)	0 (0)	0 (0)	
	It depends on the child’s age	19 (5.03)	5 (3.47)	5 (5.1)	3 (4.84)	6 (8.11)	
	NA	3 (0.79)	1 (0.69)	2 (2.04)	0 (0)	0 (0)	
In your opinion, the word “fever” describes what temperature?	It depends on the child’s age	19 (5.03)	10 (6.94)	5 (5.1)	1 (1.61)	3 (4.05)	0.257
	**Axillary ≥ 37.9°**	**203 (53.7)**	**74 (51.39)**	**63 (64.29)**	**32 (51.61)**	**34 (45.95)**	
	Axillary ≥ 38.5°	103 (27.25)	37 (25.69)	20 (20.41)	19 (30.65)	27 (36.49)	
	Other	51 (13.49)	22 (15.28)	11 (11.22)	9 (14.51)	9 (12.16)	
	NA	7 (1.85)	3 (2.08)	0 (0)	2 (3.23)	2 (2.7)	
What antipyretic do you administer most frequently?	**Oral paracetamol**	**311 (82.28)**	**119 (82.64)**	**87 (88.78)**	**50 (80.65)**	**55 (74.32)**	**0.022**
	Rectal paracetamol	98 (25.93)	33 (22.92)	17 (17.35)	14 (22.58)	34 (45.95)	
	**Oral ibuprofen**	**11 (2.91)**	**7 (4.86)**	**3 (3.06)**	**1 (1.61)**	**0 (0)**	
	Intravenous paracetamol	2 (0.53)	0 (0)	0 (0)	0 (0)	2 (2.7)	
	NA	1 (0.26)	1 (0.69)	0 (0)	0 (0)	0 (0)	
If you prescribe paracetamol more frequently than ibuprofen, why do you do so?	Better tolerability with equal efficacy	273 (72.99)	112 (80)	79 (80.61)	55 (88.71)	27 (36.49)	**<0.001**
	Better tolerability and greater efficacy	30 (8.02)	8 (5.71)	10 (10.2)	6 (9.68)	6 (8.11)	
	Better tolerability with less efficacy	4 (1.07)	2 (1.43)	1 (1.02)	0 (0)	1 (1.35)	
	Other	16 (4.28)	10 (7.14)	6 (6.12)	0 (0)	0 (0)	
	NA	55 (14.55)	12 (8.33)	2 (2.04)	1 (1.61)	40 (54.05)	
If you prescribe ibuprofen more frequently than paracetamol, why do you do so?	Equal tolerability and greater efficacy	3 (27.27)	2 (28.57)	1 (33.33)	0 (0)	0 (0)	1
	Better anti-inflammatory drug	1 (9.09)	1 (14.29)	0 (0)	0 (0)	0 (0)	
	NA	374 (98.94)	141 (97.91)	97 (98.97)	62 (100.0)	74 (100.0)	
What dose of paracetamol do you use?	**10–15 mg/kg/dose up to a maximum of 90**	205 (54.23)	82 (56.94)	63 (64.29)	41 (66.13)	19 (25.68)	**<0.001**
	10–15 mg/kg/dose up to a maximum of 120	29 (7.67)	17 (11.81)	6 (6.12)	4 (6.45)	2 (2.7)	
	10 mg/kg/dose per os and 20 mg/kg/dose rectally	81 (21.43)	43 (29.86)	25 (25.51)	12 (19.35)	1 (1.35)	
	Other	310 (82.01)	140 (97.22)	91 (92.86)	57 (91.94)	22 (29.73)	
	NA	51 (13.49)	1 (0.69)	6 (6.12)	4 (6.45)	40 (54.05)	
What dose of ibuprofen do you use?	5–10 mg/kg/dose up to a maximum of 60	200 (52.91)	82 (56.94)	61 (62.24)	42 (67.74)	15 (20.27)	**<0.001**
	5–10 mg/kg/dose up to a maximum of 120	16 (4.23)	8 (5.56)	6 (6.12)	1 (1.61)	1 (1.35)	
	5–10 mg/kg/dose per os up to a maximum of 20 mg/kg/dose	28 (7.41)	16 (11.11)	8 (8.16)	4 (6.45)	0 (0)	
	**5–10 mg/kg/dose up to a maximum of 30 mg/kg/dose**	10 (2.65)	0 (0)	6 (6.12)	4 (6.45)	0 (0)	
	Other	22 (5.82)	10 (6.94)	0 (0)	2 (3.23)	10 (13.51)	
	NA	104 (27.51)	28 (19.44)	19 (19.39)	9 (14.52)	48 (64.86)	
Do you suggest alternating the administration of ibuprofen and paracetamol?	**No**	**302 (79.89)**	**126 (87.5)**	**73 (74.49)**	**54 (87.1)**	**49 (66.22)**	0.090
	Yes	58 (15.34)	17 (11.81)	23 (23.47)	8 (12.9)	10 (13.51)	
	NA	18 (4.76)	1 (0.69)	2 (2.04)	0 (0)	15 (20.27)	
In your opinion, what is the main side effect of paracetamol?	Gastritis	10 (2.65)	3 (2.08)	4 (4.08)	1 (1.61)	2 (2.7)	**0.012**
	**Hepatotoxicity**	**326 (86.24)**	**119 (82.64)**	**87 (88.78)**	**58 (93.55)**	**62 (83.78)**	
	Other	28 (7.41)	19 (13.19)	5 (5.1)	3 (4.84)	1 (1.35)	
	NA	21 (5.56)	9 (6.25)	3 (3.06)	0 (0)	9 (12.16)	
In your opinion, what is the main side effect of ibuprofen?	**Gastritis or gastrointestinal bleeding**	**261 (69.05)**	**106 (73.61)**	**64 (65.31)**	**49 (79.03)**	**42 (56.76)**	**0.003**
	Renal insufficiency	87 (23.02)	27 (18.75)	23 (23.47)	15 (24.19)	22 (29.73)	
	Other	41 (10.85)	26 (18.06)	9 (9.18)	2 (3.23)	4 (5.41)	
	NA	31 (8.2)	11 (7.64)	8 (8.16)	2 (3.23)	10 (13.51)	

The correct answers according to the Italian Pediatric Society’s guidelines for the management of fever and the significant *p*-values are written in bold.

**Table 3:** Pain management by healthcare providers.

Question	Multiple Choice Answers	Total *n* = 378	Primary Care Pediatricians *n* = 144	Hospital Pediatricians *n* = 98	Pediatric Residents *n* = 62	Pediatric Nurses *n* = 74	*p*-Value
		*n* (%)	*n* (%)	*n* (%)	*n* (%)	*n* (%)	
Do you try to quantify the suffering of a child with pain?	No	21 (5.56)	12 (8.33)	2 (2.04)	6 (9.68)	1 (1.35)	0.026
	Yes	353 (93.39)	132 (91.67)	95 (96.94)	55 (88.71)	71 (95.95)	
	NA	4 (1.06)	0 (0)	1 (1.02)	1 (1.61)	2 (2.7)	
How do you quantify pain in a breastfeeding infant?	Intensity of crying	12 (3.17)	7 (4.86)	2 (2.04)	0 (0)	3 (4.05)	0.214
	Position of legs	4 (1.06)	0 (0)	1 (1.02)	0 (0)	3 (4.05)	
	Facial expression	5 (1.32)	3 (2.08)	2 (2.04)	0 (0)	0 (0)	
	Degree of agitation	4 (1.06)	2 (1.39)	1 (1.02)	1 (1.61)	0 (0)	
	Multiple behavioural evaluations	347 (91.8)	131 (90.97)	90 (91.84)	60 (96.77)	66 (89.19)	
	NA	6 (1.59)	1 (0.69)	2 (2.04)	1 (1.61)	2 (2.7)	
How do you quantify pain in a child aged 3–4 years?	On the basis of what he/she manages to report	34 (8.99)	17 (11.81)	10 (10.2)	3 (4.84)	4 (5.41)	0.015
	On the basis of his/her behaviour	104 (27.51)	47 (32.64)	26 (26.53)	15 (24.19)	16 (21.62)	
	Intensity of crying	21 (5.56)	14 (9.72)	2 (2.04)	1 (1.61)	4 (5.41)	
	Using visual scales	260 (68.78)	91 (63.19)	68 (69.39)	51 (82.26)	50 (67.57)	
	NA	9 (2.38)	2 (1.39)	1 (1.02)	1 (1.61)	5 (6.76)	
What does the choice of pain therapy you recommend depend on?	The intensity of the pain	272 (71.96)	104 (72.22)	71 (72.45)	49 (79.03)	48 (64.86)	0.036
	The child’s age	66 (17.46)	27 (18.75)	11 (11.22)	12 (19.35)	16 (21.62)	
	The duration of the pain	27 (7.14)	13 (9.03)	3 (3.06)	5 (8.06)	6 (8.11)	
	The origin of the pain	109 (28.84)	39 (27.08)	30 (30.61)	21 (33.87)	19 (25.68)	
	NA	14 (3.7)	3 (2.08)	1 (1.02)	1 (1.61)	9 (12.16)	
What drug do you use to treat mild-moderate pain?	Paracetamol	332 (87.83)	126 (87.5)	86 (87.76)	59 (95.16)	61 (82.43)	<0.001
	Paracetamol + codeine	9 (2.38)	4 (2.78)	4 (4.08)	0 (0)	1 (1.35)	
	Ibuprofen	44 (11.64)	26 (18.06)	13 (13.27)	4 (6.45)	1 (1.35)	
	Sedative	1 (0.26)	0 (0)	1 (1.02)	0 (0)	0 (0)	
	NA	15 (3.97)	1 (0.69)	1 (1.02)	1 (1.61)	12 (16.22)	

The correct answers according to the Italian Pediatric Society’s guidelines for the management of pain and the significant *p*-values are written in bold.

**Table 4:** Demographic characteristics of responding parents.

Question	Multiple Choice Answers	Total *n* = 464	Age of Youngest or Only Child 0–5 Years *n* = 175 (37.7%)	Age of Youngest or Only Child 6–10 Years *n* = 175 (37.7%)	Age of Youngest or Only Child 11–14 Years *n* = 114 (24.6%)	*p*-Value
		*n* (%)	*n* (%)	*n* (%)	*n* (%)	
Median age (min–max)		41 (20, 56)	37 (20–54)	42 (28–55)	47 (35–56)	<0.001
Gender	Male	91 (19.61)	34 (19.43)	38 (21.71)	19 (16.67)	0.601
	Female	373 (80.39)	141 (80.57)	137 (78.29)	95 (83.33)	
Where do you live?	Principal city	309 (66.59)	125 (71.43)	119 (68)	65 (57.02)	0.099
	Industrial municipality	105 (22.63)	31 (17.71)	39 (22.29)	35 (30.7)	
	Rural municipality	50 (10.78)	19 (10.86)	17 (9.71)	14 (12.28)	
Ethnic origin	Caucasian	433 (93.32)	159 (90.86)	162 (92.57)	112 (98.25)	0.075
	Middle Eastern	6 (1.29)	2 (1.14)	3 (1.71)	1 (0.88)	
	Hispanic	18 (3.88)	8 (4.57)	9 (5.14)	1 (0.88)	
	Oriental	7 (1.51)	6 (3.43)	1 (0.57)	0 (0)	
Education	Middle school	57 (12.28)	26 (14.86)	18 (10.29)	13 (11.4)	0.531
	High school	199 (42.89)	73 (41.71)	72 (41.14)	54 (47.37)	
	University	208 (44.83)	76 (43.43)	85 (48.57)	47 (41.23)	
Occupation	Unemployed	38 (8.19)	17 (9.71)	16 (9.14)	5 (4.39)	0.919
	Housewife	48 (10.34)	17 (9.71)	16 (9.14)	15 (13.16)	
	Craftsman/woman	6 (1.29)	3 (1.71)	2 (1.14)	1 (0.88)	
	Blue collar worker	38 (8.19)	15 (8.57)	14 (8)	9 (7.89)	
	White collar worker	217 (46.77)	84 (48)	81 (46.29)	52 (45.61)	
	Manager	26 (5.6)	10 (5.71)	9 (5.14)	7 (6.14)	
	Self-employed	90 (19.4)	29 (16.57)	36 (20.57)	25 (21.93)	
	NA	1 (0.22)	0 (0)	1 (0.57)	0 (0)	
Number of children	1	251 (66.58)	70 (40)	51 (29.14)	27 (23.68)	<0.001
	2	125 (33.16)	84 (48)	98 (56)	50 (43.86)	
	3 or more	1 (0.27)	21 (12)	26 (14.86)	37 (32.46)	

In the case of the categorical variables, the table shows absolute frequencies with percentage frequencies in brackets. NA: not available.

**Table 5:** Parental management of fever.

Question	Multiple Choice Answers	Total *n* = 464	Age of Youngest or Only Child 0–5 Years *n* = 175 (37.7%)	Age of Youngest or Only Child 6–10 Years *n* = 175 (37.7%)	Age of Youngest or Only Child 11–14 Years *n* = 114 (24.6%)	*p*-Value
		*n* (%)	*n* (%)	*n* (%)	*n* (%)	
How do you see fever?	A sign of disease that should always be eliminated	36 (7.76)	11 (6.29)	13 (7.43)	12 (10.53)	0.514
	A sign of disease that should only be eliminated if high	151 (32.54)	55 (31.43)	63 (36)	33 (28.95)	
	A bodily defence mechanism	277 (59.7)	109 (62.29)	99 (56.57)	69 (60.53)	
What type of thermometer do you use to measure your child’s temperature?	Mercury	104 (22.41)	14 (8)	47 (26.86)	43 (37.72)	<0.001
	Infrared	33 (7.11)	19 (10.86)	13 (7.43)	1 (0.88)	
	Liquid crystals liquid	43 (9.27)	6 (3.43)	21 (12)	16 (14.04)	
	Electronic	284 (61.21)	136 (77.71)	94 (53.71)	54 (47.37)	
Where do your measure your child’s temperature?	Rectum	97 (20.91)	73 (41.71)	19 (10.86)	5 (4.39)	<0.001
	Armpit	305 (65.73)	72 (41.14)	131 (74.86)	102 (89.47)	
	Ear	28 (6.03)	14 (8)	11 (6.29)	3 (2.63)	
	Forehead	32 (6.9)	15 (8.57)	13 (7.43)	4 (3.51)	
	Other	2 (0.43)	1 (0.57)	1 (0.57)	0 (0)	
In your opinion, the word “fever” describes what temperature?	It depends on the child’s age	12 (2.59)	6 (3.43)	2 (1.14)	4 (3.51)	0.108
	Axillary ≥ 36.5°	1 (0.22)	1 (0.57)	0 (0)	0 (0)	
	Axillary ≥ 37°	94 (20.26)	32 (18.29)	37 (21.14)	25 (21.93)	
	Axillary ≥ 37.5°	163 (35.13)	53 (30.29)	59 (33.71)	51 (44.74)	
	Axillary ≥ 37.9°	139 (29.96)	59 (33.71)	54 (30.86)	26 (22.81)	
	Axillary ≥ 38.5°	55 (11.85)	24 (13.71)	23 (13.14)	8 (7.02)	
	>39	10 (2.16)	4 (2.29)	2 (1.14)	4 (3.51)	
Why do you think it is necessary to treat fever?	Because it is associated with headache and ill-being, it is better to eliminate it	221 (47.63)	71 (40.57)	79 (45.14)	71 (62.28)	<0.001
	Fever can have serious consequences	204 (43.97)	81 (46.29)	84 (48)	39 (34.21)	
	By eliminating fever I am treating the disease that caused it	39 (8.41)	23 (13.14)	12 (6.86)	4 (3.51)	
If you think that fever can have serious consequences, which do you fear the most?	Convulsions	350 (75.43)	134 (76.57)	129 (73.71)	87 (76.32)	0.345
	Damage to the nervous system	59 (12.72)	21 (12)	28 (16)	10 (8.77)	
	Dehydration	45 (9.7)	15 (8.57)	16 (9.14)	14 (12.28)	
	Coma	2 (0.43)	1 (0.57)	0 (0)	1 (0.88)	
	Death	5 (1.08)	1 (0.57)	2 (1.14)	2 (1.75)	
	None of the above	3 (0.65)	3 (1.71)	0 (0)	0 (0)	
When treating a child with fever, what is the first thing to do?	Reduce the number of clothes	199 (42.89)	91 (52)	64 (36.57)	44 (38.6)	0.059
	Put an ice pack on his/her head	11 (2.37)	2 (1.14)	5 (2.86)	4 (3.51)	
	Put a cloth soaked in cold water on his/her forehead	63 (13.58)	20 (11.43)	27 (15.43)	16 (14.04)	
	Put a cloth soaked in lukewarm water on his/her forehead	3 (0.65)	1 (0.57)	0 (0)	2 (1.75)	
	Sponge all of his/her body with alcohol	2 (0.43)	1 (0.57)	0 (0)	1 (0.88)	
	Administer an antipyretic	186 (40.09)	60 (34.29)	79 (45.14)	47 (41.23)	
If you use an antipyretic, do you consult your pediatrician before administering it?	No	270 (58.19)	92 (52.57)	100 (57.14)	78 (68.42)	0.027
	Yes	194 (41.81)	83 (47.43)	75 (42.86)	36 (31.58)	
If you use an antipyretic, do you also use physical means to reduce your child’s temperature?	No	208 (44.83)	91 (52)	76 (43.43)	41 (35.96)	0.025
	Yes	256 (55.17)	84 (48)	99 (56.57)	73 (64.04)	
If so, what do you use?	Ice pack	57 (22.27)	9 (10.71)	30 (30.3)	18 (24.66)	0.025
	Cloth soaked in cold water	170 (66.41)	66 (78.57)	58 (58.59)	46 (63.01)	
	Cloth soaked in lukewarm water	23 (8.98)	7 (8.33)	10 (10.1)	6 (8.22)	
	Sponging with alcohol	6 (2.34)	2 (2.38)	1 (1.01)	3 (4.11)	
	NA	*208*	*91*	*76*	*41*	
Which antipyretic do you give your child most frequently?	Paracetamol	409 (88.15)	156 (89.14)	148 (84.57)	105 (92.11)	0.066
	Ibuprofen	54 (11.64)	19 (10.86)	27 (15.43)	8 (7.02)	
	Aspirin	1 (0.22)	0 (0)	0 (0)	1 (0.88)	
If you use paracetamol more frequently than ibuprofen, why do you do so?	Better tolerability with equal efficacy	254 (62.1)	95 (60.9)	91 (61.49)	68 (64.76)	0.079
	Better tolerability and greater efficacy	46 (11.25)	17 (10.9)	19 (12.84)	10 (9.52)	
	Recommended by paediatrician	41 (10.02)	23 (14.74)	14 (9.46)	4 (3.81)	
	Habit	68 (16.63)	21 (13.46)	24 (16.22)	23 (21.9)	
	NA	*54*	*19*	*27*	*8*	
If you use ibuprofen more frequently than paracetamol, why do you do so?	Better tolerability with equal efficacy	15 (27.78)	6 (31.58)	8 (29.63)	1 (12.5)	0.335
	Equal tolerability and greater efficacy	37 (68.52)	11 (57.89)	19 (70.37)	7 (87.5)	
	Recommended by pediatrician	2 (3.7)	2 (10.53)	0 (0)	0 (0)	
	NA	*409*	*156*	*148*	*105*	
Which way of administering paracetamol do you prefer?	**Oral**	**305 (65.73)**	**88 (50.29)**	**103 (58.86)**	**114 (100)**	**<0.001**
	Rectal	158 (34.05)	86 (49.14)	72 (41.14)	0 (0)	
	NA	1 (0.22)	1 (0.57)	0 (0)	0 (0)	
Which paracetamol formulation do you prefer?	**Syrup**	**195 (42.03)**	**67 (38.29)**	**82 (46.86)**	**46 (40.35)**	**<0.001**
	**Drops**	**30 (6.47)**	**19 (10.86)**	**8 (4.57)**	**3 (2.63)**	
	Suppositories	158 (34.05)	86 (49.14)	72 (41.14)	0 (0)	
	**Orally dispersible granulate**	**57 (12.28)**	**3 (1.71)**	**11 (6.29)**	**43 (37.72)**	
	**Tablets**	**22 (4.74)**	**0 (0)**	**2 (1.14)**	**20 (17.54)**	
	NA	2 (0.43)	0 (0)	0 (0)	2 (1.75)	
Do you alternate the administration of ibuprofen and paracetamol?	**No**	**405 (87.28)**	**162 (92.57)**	**146 (83.43)**	**97 (85.09)**	**0.021**
	You	59 (12.72)	13 (7.43)	29 (16.57)	17 (14.91)	
What do you think is the main side effect of paracetamol?	None	296 (72.37)	114 (73.08)	112 (75.68)	70 (66.67)	0.332
	Gastritis	23 (5.62)	6 (3.85)	9 (6.08)	8 (7.62)	
	Hives	10 (2.44)	8 (5.13)	2 (1.35)	0 (0)	
	Liver toxicity	60 (14.67)	17 (10.9)	20 (13.51)	23 (21.9)	
	Diarrhea	19 (4.65)	11 (7.05)	5 (3.38)	3 (2.86)	
	NA	1 (0.24)	0 (0)	0 (0)	1 (0.95)	
What do you think is the main side effect of ibuprofen?	**Gastritis or gastrointestinal bleeding**	**23 (42.59)**	**8 (42.11)**	**13 (48.15)**	**2 (25)**	**0.022**
	Renal insufficiency	2 (3.7)	2 (10.53)	0 (0)	0 (0)	
	Bronchospasm	1 (1.85)	0 (0)	1 (3.7)	0 (0)	
	Skin rash	2 (3.7)	2 (10.53)	0 (0)	0 (0)	
	None	21 (38.89)	7 (36.84)	11 (40.74)	3 (37.5)	
	NA	5 (9.26)	0 (0)	2 (7.41)	3 (37.5)	

NA: not available.

**Table 6:** Parental management of pain.

Question	Multiple Choice Answers	Total *n* = 464	Age of Youngest or Only Child 0–5 Years *n* = 175 (37.7%)	Age of Youngest or Only Child 6–10 Years *n* = 175 (37.7%)	Age of Youngest or Only Child 11–14 Years *n* = 114 (24.6%)	*p*-Value
		*n* (%)	*n* (%)	*n* (%)	*n* (%)	
Do you think that pain is educationally useful for the growth of your child and that, for this reason, he/she should learn to bear it?	No	229 (49.35)	97 (55.43)	79 (45.14)	53 (46.49)	0.121
	Yes	235 (50.65)	78 (44.57)	96 (54.86)	61 (53.51)	
Do you think it is possible to understand when even a young child is feeling pain and how great it is?	No	140 (30.17)	55 (31.43)	50 (28.57)	35 (30.7)	0.848
	Yes	324 (69.83)	120 (68.57)	125 (71.43)	79 (69.3)	
In the case of mild-moderate pain, what is your first-choice drug?	Paracetamol	363 (78.23)	136 (77.71)	132 (75.43)	95 (83.33)	0.424
	Paracetamol + codeine	2 (0.43)	1 (0.57)	0 (0)	1 (0.88)	
	Ibuprofen	47 (10.13)	17 (9.71)	23 (13.14)	7 (6.14)	
	Sedative	1 (0.22)	0 (0)	1 (0.57)	0 (0)	
	Nothing	51 (10.99)	21 (12)	19 (10.86)	11 (9.65)	
